# Supplementary material for: In Silico Characterisation of Putative Prophages in Lactobacillaceae Used in Probiotics for Vaginal Health
Source: Microorganisms. 2022 Jan 20;10(2):214. doi: 10.3390/microorganisms10020214 (PMC8879116; doi:10.3390/microorganisms10020214)
Supplement: Supplementary file 1 [file microorganisms-10-00214-s001.zip › microorganisms-1498226 Supplementary Revision/Supplementary Fig_15Dec2021.pdf]

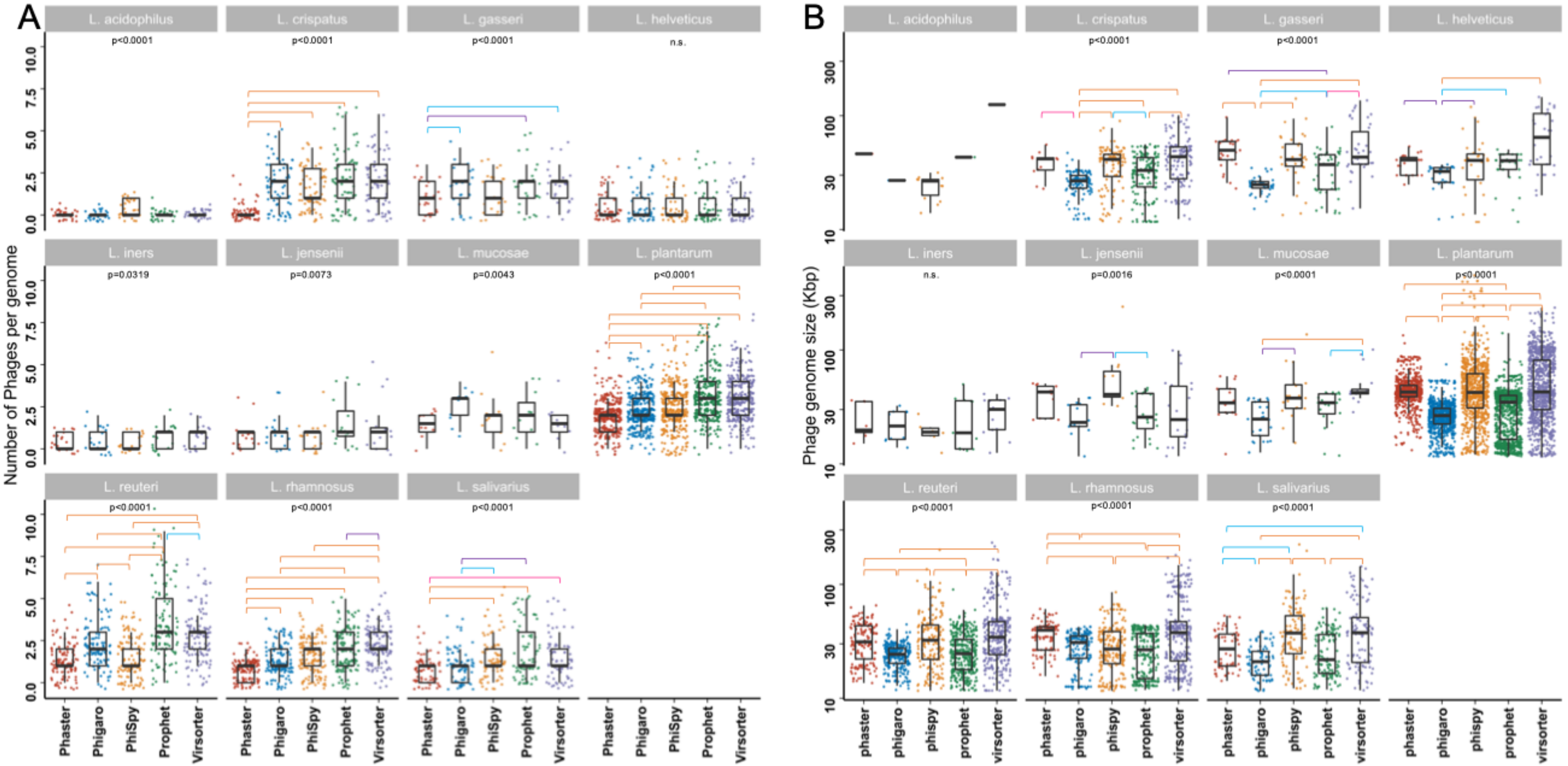

Figure S1. Putative prophages detected by various detection algorithm. The number (A) and size (B) of putative prophage sequences detected using Phaster, Phigaro, Phispy, Prophet and Virsorter in publicly available genomes of *L. acidophilus* (n = 30), *L. crispatus* (n = 70), *L. gasseri* (n = 25), *L. helveticus* (n = 49), *L. iners* (n = 19), *L. jensenii* (n = 16), *L. mucosae* (n = 10), *L. plantarum* (n = 344), *L. reuteri* (n = 116), *L. rhamnosus* (n = 139), and *L. salivarius* (n = 77). ANOVA was used to test for differences between algorithms. The color-coded brackets indicate significance levels after adjusting for multiple comparisons: $p<0.05$ = blue bracket, $p<0.01$ = purple bracket, $p<0.001$ = pink bracket, $p<0.0001$ =orange bracket.

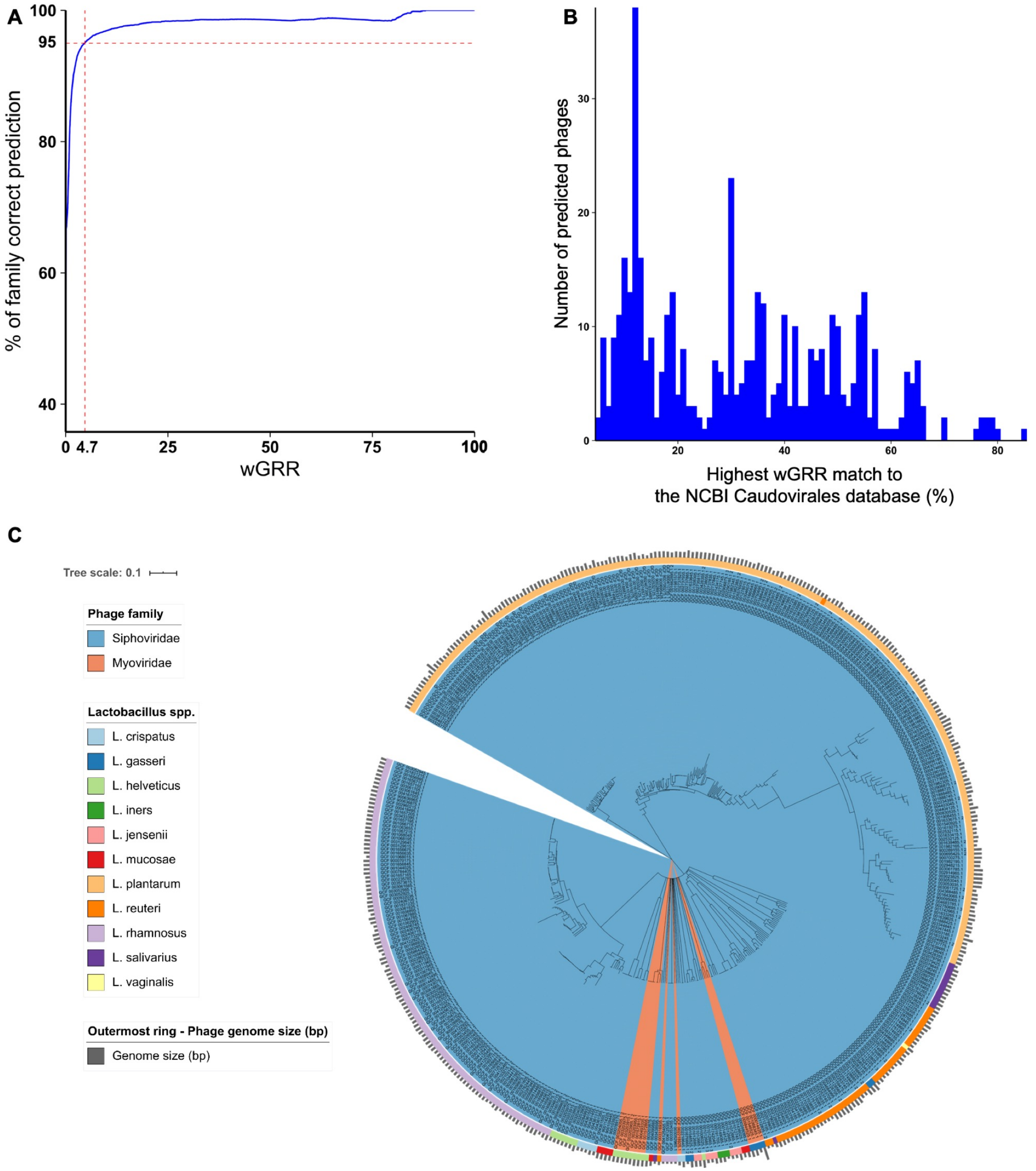

Figure S2. Weighted gene repertoire relatedness (wGRR). To assign putative bacteriophages to families, (A) a pairwise wGRR similarity matrix for all bacteriophages in the NCBI *Caudovirales* database was calculated, indicating that bacteriophages with a wGRR similarity score of 4.7 or higher belonged to the same family with ≥95% prediction correctness. (B) shows the highest hits wGRR match of predicted bacteriophages to bacteriophages in the NCBI *Caudovirales* database. (C) Average nucleotide identity (ANI) between all predicted bacteriophages with families assigned using the wGRR approach. The second to last ring shows the *Lactobacillaceae* spp. host of the predicted bacteriophages and the outermost indicates phage genome size.

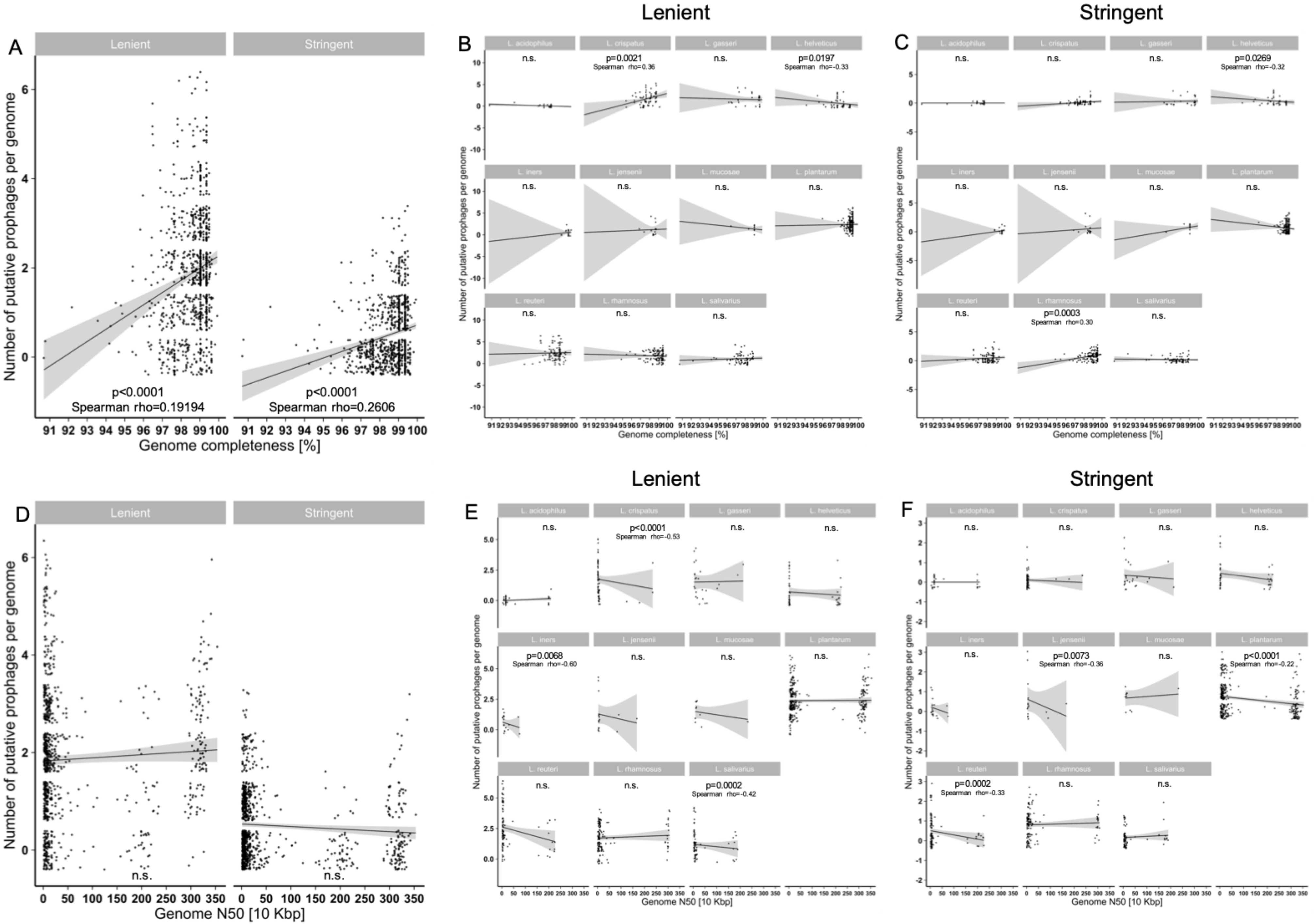

Figure S3. Correlation of the number of predicted putative prophages with genome quality measures. Overall genome completeness (A), genome completeness by *Lactobacillaceae* spp. (B), overall genome N50 (C) and genome N50 by *Lactobacillaceae* spp. (D) were correlated with the number of putative prophages predicted per genome. Regression line and confidence intervals were fitted using a liner model and Spearman rank correlation coefficients are shown. n.s. corresponds to not significant (p>0.05).

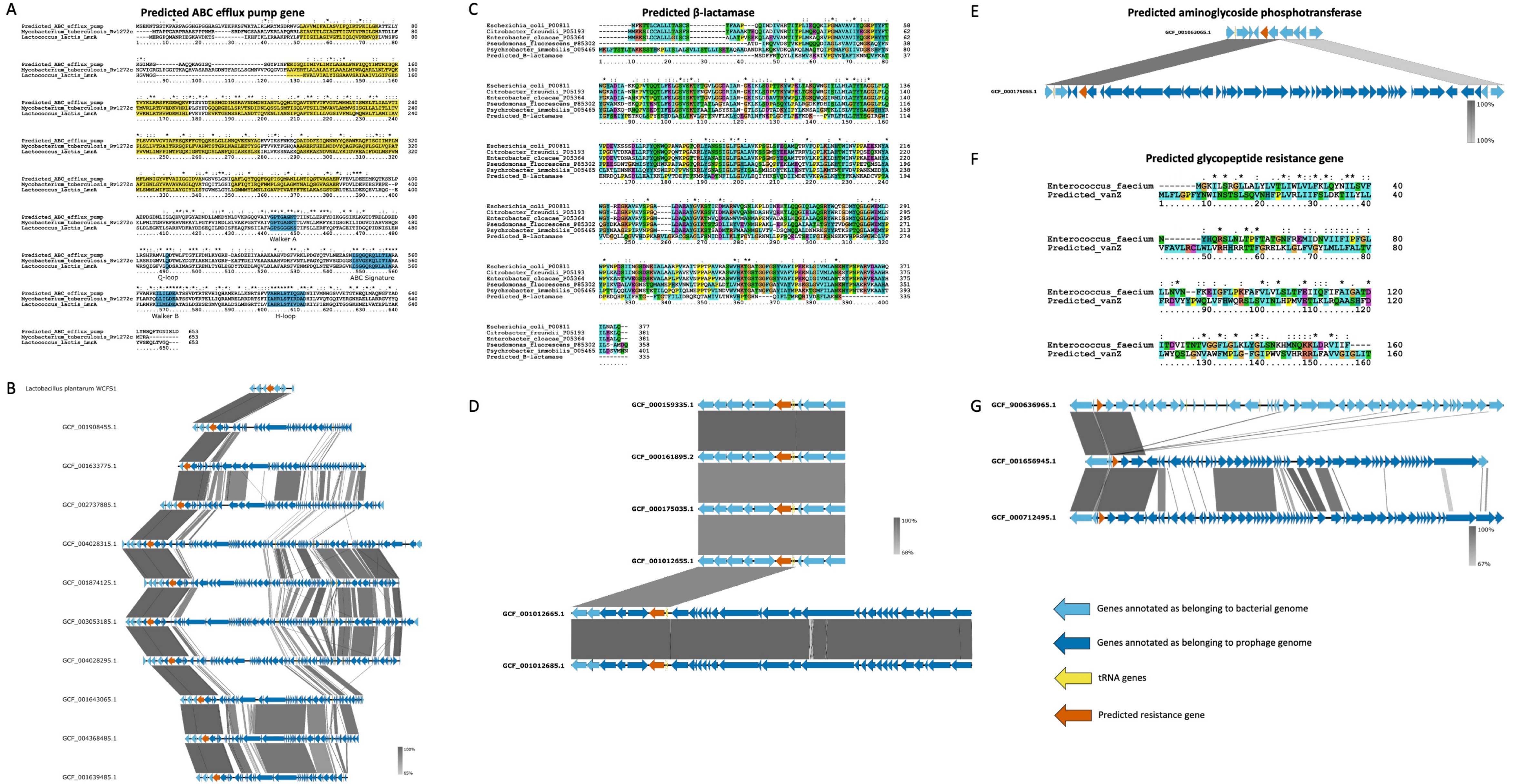

Figure S4. Putative bacteriophage-encoded β-lactamase and ABC efflux pump. Amino-acid pileup of (A) the putative ABC efflux pump and previously characterised efflux pumps in *Mycobacterium tuberculosis* (Rv1272c) and *Lactococcus lactis* (LmrA), (C) the putative β-lactamase and the five closest related functionally characterised proteins, and (F) the putative glycopeptide resistance gene VanZ and previously characterised functional VanZ from *Enterococcus faecium*. The genomic context for (B) the putative ABC efflux pump in one *L. plantarum* strain without and the ten strains with a predicted bacteriophage, (D) the putative β-lactamase in four *L. jensenii* strains without and two strains with the predicted phage, (E) the putative aminoglycoside phosphotransferase in one *L. gasseri* strain with and without predicted phage are shown, and (G) the putative glycopeptide resistance VanZ in one *L. rhamnosus* strain without and two strains with the predicted bacteriophage are shown.
